# What is the impact of sex hormones on the pathogenesis of rheumatoid arthritis?

**DOI:** 10.3389/fmed.2022.909879

**Published:** 2022-07-22

**Authors:** Charles Raine, Ian Giles

**Affiliations:** Centre for Rheumatology, Department of Inflammation, Division of Medicine, University College London, London, United Kingdom

**Keywords:** rheumatoid arthritis, estrogen, progesterone, androgens, pathogenesis, pregnancy

## Abstract

Rheumatoid arthritis (RA) is the most common inflammatory rheumatic disease and has a female predominance of around 3:1. The relationship between sex hormones and RA has been of great interest to researchers ever since Philip Hench's observations in the 1930's regarding spontaneous disease amelioration in pregnancy. Extensive basic scientific work has demonstrated the immunomodulatory actions of sex hormones but this therapeutic potential has not to date resulted in successful clinical trials in RA. Epidemiological data regarding both endogenous and exogenous hormonal factors are inconsistent, but declining estrogen and/or progesterone levels in the menopause and post-partum appear to increase the risk and severity of RA. This review assimilates basic scientific, epidemiological and clinical trial data to provide an overview of the current understanding of the relationship between sex hormones and RA, focusing on estrogen, progesterone and androgens.

## Introduction

Rheumatoid arthritis (RA) is a chronic multisystem inflammatory disease which causes a destructive symmetrical polyarthritis. It is characterized by production of the autoantibodies rheumatoid factor (RF) and anti-citrullinated protein antibodies (ACPA). Common to many autoimmune disorders, there is a female predominance, with a female:male distribution of around 3:1 (1), although this gender disparity is less marked than in other inflammatory rheumatic conditions such as systemic lupus erythematosus. Other observations implicating the importance of sex hormones in the pathogenesis of RA include its peak incidence at menopause (2), reduced disease activity in pregnancy and flare in the postpartum period (3). Such phenomena would suggest that declining female sex hormones, principally oestrogren and progesterone, increase the risk of RA in menopause and post-partum, while increased levels in pregnancy are protective. However, despite extensive study, the relationship between sex hormones and RA pathogenesis remains complex.

This review will synthesize lines of evidence regarding the impact of sex hormones on the pathogenesis of RA, focusing on estrogen, progesterone and androgens. We present an overview of the data from basic laboratory research through to a comprehensive evaluation of epidemiological studies and clinical trials.

## Strategy

We searched PubMed for original articles, systematic reviews and meta-analyses in English, pubished between 1 January 1990 and 31 December 2021. Reference lists in retrieved articles were also reviewed to identify further articles of relevance. Search terms used comprised combinations of the following: “Rheumatoid arthritis”, “sex hormones/steroids”, “(o)estrogen”, “progesterone”, “androgens”, “risk factors”, “disease activity”, “menarche”, “pregnancy”, “menopause”, “contraceptive”, “breastfeeding” and “hormone replacement therapy”.

## Rheumatoid arthritis pathogenesis

A detailed discussion of the pathogenesis of rheumatoid arthritis it outside the scope of this article, and the reader is directed to other recent excellent reviews (4–7). In brief, the etiology of RA is recognized to depend on complex interactions between genes and environment, with a resultant breakdown of immune tolerance leading to inflammation in synovial joints. The strongest genetic component is found in the human leukocyte antigen (HLA) class II molecule HLA-DRB1, with over 80 % of patients carrying the shared epitope of the HLA-DRB1^*^04 cluster (8), which is also associated with severity of disease (9). RA pathogenesis is thought to be initiated years before the development of symptoms and likely involves the induction by environmental factors, the most established of which is smoking (10), of post-translational modifications. These processes lead to the activation by antigen-presenting cells of an adaptive immune response, with production of the hallmark autoantibodies RF (targeting immunoglobulin G) and ACPA (6). Interactions of immune cells, including T and B cells, plasma cells and monocytes, and pro-inflammatory cytokines, most importantly tumor necrosis factor-alpha (TNF-α) and interleukin (IL)-6, lead to an infux and local activation of inflammatory cells in the synovium. Macrophage-like synoviocytes proliferate and produce TNF-α, IL-6 and IL-1, among other pro-inflammatory mediators. Infiltrating immune cells include CD4+ T cells and mature B cells producing RF and ACPAs. The synovial membrane expands and is filled with new blood vessels. Within the synovium, abundant osteoclasts are the primary mediators of bone erosion, while fibroblast-like synoviocytes secrete matrix metalloproteinases into the synovial fluid leading to cartilage degradation.

## Sex hormone signaling

The sex hormones estrogen, progesterone and androgens all bind to nuclear receptors which belong to the type 1 family of nuclear hormone transcription factors (to which the glucocorticoid receptor also belongs) (11–13). In brief, binding of these hormones to their receptors in the cytosol leads to dissociation of heat shock proteins, homodimerization, translocation into the nucleus and subsequent binding to hormone response elements within regulatory regions of target genes, resulting in modification of gene transcription. Classically, oestrogens bind the cytosolic receptors estrogen receptor (ER)- α and/or ERβ to mediate genomic effects; in addition, oestrogens may also bind to membrane receptors such as G protein-coupled estrogen receptor 1 (GPER1) to trigger downstream signaling cascades that non-directly influence gene expression. The progesterone receptor (PR) and androgen receptor (AR), which are closely related, mediate classical genomic effects in a similar fashion to oestrogens, with hormone-receptor complexes binding to progresterone- and androgen-response elements respectively.

## Sex hormones and genetic risk of rheumatoid arthritis

### Estrogen and progesterone

Genome-wide and targeted gene analysis studies have revealed single nucleotide polymorphisms (SNPs) in sex steroid-associated genes that influence the risk and course of RA. These include SNPs in *ESR2* which encodes estrogen receptor- β (ERβ) conferring a reduced risk of erosive arthritis (14) and an improved chance of responding to anti-tumor necrosis factor (TNF) therapy (15); other SNPs associated with a reduced risk of erosive disease include those in cytochrome p450 enzymes CYP1B1 and CYP2C9, which convert oestrogens to anti-inflammatory hydroxy-oestrogens (14). We are not aware of any studies evaluating genes involved in progesterone signaling and the risk or severity of RA.

### Androgens

SNPs in the cytochrome B5-encoding gene *CYB5A* have been associated with a reduced susceptibility to RA in women (16). Such polymorphisms lead to an increased production by the cytochrome B5 enzyme of androgens from precursor hormones, suggesting that higher androgen levels protect against the development of RA.

## Sex hormone signaling in the immune system

There is a significant body of evidence of the immunomodulatory effects mediated by sex hormones in autoimmune disease [extensively reviewed in Hughes (17), Wilder and Elenkov (18), Hughes and Choubey (19), Kanik and Wilder (20), Cutolo and Straub (21) and Moulton (22)].

### Estrogen

Oestrogens have complex interactions with the immune system which may be pro- or anti-inflammatory depending on the cell type and concentrations involved (21, 22). With relevance to B cell function, at physiological concentrations the estrogen-ERα complex binds to the promoter of the *AICDA* gene which in turn stimulates expression of the enzyme activation-induced cytidine deaminase (AID). This enzyme is the master regulator of somatic hypermutation and class switch recombination. Therefore, oestrogens support immunoglobulin class switching in B cells and would logically be expected to have a deleterious effect on autoimmune diseases characterized by autoantibody production (23).

T cell ontogeny comprises initial development as haematopoetic precusors in the bone-marrow which then migrate to the thymus. Here, a process of positive and negative selection occurs, such that T cells which react strongly with antigen presented by major histocompatibility complex survive, but those that react with self-antigen are eliminated (except a proportion of CD4+ cells that survive as regulatory T cells (T_reg_), see below). The process of negative selection is regulated by the transcription factor AIRE, which serves to promote self-tolerance. This protein has emerged as a key factor in sexual dimorphism in autoimmunity, with females expressing lower levels of AIRE post-puberty compared to males (24). Male castration reduces expression of AIRE, while ERα deficient mice showed no difference in expression between sexes. Moreover, estrogen treatment was found to decrease AIRE expression in human thymic tissue *via* methylation of CpG sites in the *AIRE* promoter (24).

In contrast, oestrogens have been found to have a range of direct anti-inflammatory actions on T cells. Silibinin, a natural agonist of ERβ was shown to reduce the *in-vitro* expression of pro-inflammatory interleukin (IL)-17 and TNFα in T cells from healthy donors and patients with active RA (25). This effect was mediated *via* down modulation of the expression of the epigenetic modifier microRNA-155. Silibinin has also been shown to stimulate apoptosis of human RA synoviocytes *in vitro* (26) and reduce the production of inflammatory cytokines in rats with collagen-induced arthritis (26).

Oestrogens generally exert inhibitory effects on pro-inflammatory T_H_1 cells, while they may inhibit pro-inflammatory T_H_17 cells *via* ERα or have the opposite effect *via* ERβ. At high concentrations such as seen in pregnancy, oestrogens induce the secretion of IL-10 and suppress TNFα production in T cells, supporting an anti-inflammatory cytokine milieu (27).

Forkhead box (FOXP3) expressing T_reg_ cells are highly suppressive regulators of the immune response through secretion of anti-inflammatory cytokines such as IL-10 as well as cell-cell contact mechanisms. The *FOXP3* locus possesses sex steroid response elements enabling direct binding of hormones and subsequent modulation of FOXP3 activation. Endogenous complexes of estradiol and ERβ were shown to bind directly and activate the FOXP3 promoter in T_reg_ cells from both human cervical cancer specimens and healthy controls (28).

It has been shown that estrogen treatment before immunization with collagen can retard disease development in collagen-induced arthritis (CIA), a mouse model of RA (29). In CIA, exogenous estrogen administration ameliorated postpartum flare (30), while joint inflammation improved with treatment of non-pregnant arthritis-prone mice with estradiol or progesterone at pregnancy-like levels (31), or by treatment with high-potency estrogen alone (32). Estradiol has been linked to expansion of anti-inflammatory T_reg_ cells in pregnant mice with autoimmune encephalitis (33) and pregnancy-induced amelioration of joint inflammation has been achieved by treatment of non-pregnant SKG mice (a model of human RA) with either estradiol or progesterone at pregnancy-like levels (31).

### Progesterone

In contrast to the dichotomous effects of oestrogens, progesterone exhibits broad anti-inflammatory actions (17, 21), including: inhibition of AID (in opposition to the effect of oestrogens); inhibition of the T_H_1 and T_H_17 response; and inhibition of NK cells, neutrophils and macrophages.

Progesterone promotes the T_H_2 response by enhancing IL-4 and IL-10 production in human T cells (27). During pregnancy, lymphocytes express progesterone receptors and release a protein named progesterone-induced blocking factor, which has strong anti-natural killer (NK) cell activity, and also secrete IL-10 (34). Progesterone has been shown to induce FOXP3+ T_regs_ from naive murine CD4+ T cells *via* suppression of mammalian target of rapamycin (mTOR) signaling (35); furthermore, progesterone promotes differentiation of human fetal cord blood T cells into T_regs_ and suppresses their differentiation to T_H_17 cells (36). In multiple sclerosis, T_regs_ express high levels of estrogen and progesterone receptors, and each hormone enhances the suppressive function of T_regs_ *in vitro* (37).

Recently, progesterone was shown to suppress activation (as measured by CD69 and CD25 expression) of *ex vivo* CD4+ T cells from healthy human females in a dose-dependent fashion (using doses similar to those found in the placenta) (38); furthermore, RNA sequencing analysis showed significant transcriptomic changes involving downregulation of immune-related genes and pathways important in RA, such as signal transducer and activator of transcription (STAT)-1 and STAT3.

### Androgens

Androgens such as testosterone have a range of anti-inflammatory effects *in vivo*, reducing secretion of inflammatory cytokines such as TNF, IL-1 and IL-6 by monocytes and inhibiting B cell lymphopoiesis and antibody production (39). In inflammatory rheumatic diseases serum androgen levels are often reduced (40) owing to the stimulation by inflammatory cytokines such as TNF, IL-1 and IL-6 of the aromatase enzyme in immune cells and fibroblasts. Androgens are also known to bind and upregulate tyrosine-protein phosphatase non-receptor type 1 (PTPN1), which has a broad range of functions in cell growth and immune function. PTPN1 inhibits janus kinase (JAK)-2 and tyrosine kinase 2, part of the JAK-STAT pathway which is integral to T_H_1 cell-mediated immune responses and the production of IL-12 and IFNγ. This signaling pathway is of especial interest in RA given the established therapeutic options targeting the JAK-STAT pathway. It is not yet known whether response to these treatments differs between men and women with RA.

Conversely to oestrogens, androgens promote AIRE transcription by recruiting androgen receptors to *AIRE* promoter regions, leading to higher expression in mice and human thymus in males compared to females (41). Male sex and androgen treatment were protective in a mouse model of multiple sclerosis, but to date this effect has not been investigated in models of rheumatic diseases including RA (41).

Similarly to oestrogens, androgens can bind to the FOXP3 androgen response element, leading to acetylation of histone H4 and activation of FOXP3; there is a strong androgen-dependent increase of FOXP3 expression in T cells from women in the ovulatory phase of the menstrual cycle but not from men (42).

## Sex hormones and pathogenesis of RA: Epidemiology

There is extensive epidemiological data implicating the importance of sex hormones in the pathophysiology of RA, but results are conflicting. Large modern cohort studies and meta-analyses have often contradicted the findings of early smaller retrospective studies. We summarize the results of large modern epidemiological studies (case-control, cohort studies and meta-analyses) concerning sex hormones and RA in Tables 1–3 (43–64). Table 1 shows studies which found an association between endogenous hormonal factors and risk or course of RA, Table 2 lists the reports which found no such association, and Table 3 shows studies concerning exogenous hormonal factors and RA (Table 3) (nb, some studies are listed more than once where appropriate).

**Table 1:** Epidemiological studies showing an association between endogenous sex hormones and rheumatoid arthritis pathogenesis.

**Reference**	**Design**	**Population/country**	**N (Total/RA)**	**Hormonal factor**	**Summary of findings (95 % CI)**
Karlson et al. (43)	Prospective cohort	Nurses aged 30–55 (at baseline), USA	121,700/674	Menarche, BF	↑ risk of RA with irregular menstrual cycles, RR 1.4 (1.0–2.0) Early menarche (<10 years)↑ risk of seropositive RA, RR 1.6 (1.1–2.4) ↓ risk of RA with ↑ duration BF, RR 0.5 (0.3–0.8) for >24 months
Pedersen et al. (44)	Case-control	Women aged 18–65, Denmark	1,284/515	Menarche	Menarche at age ≥15 years ↑ risk of ACPA-positive and -negative RA vs. menarche at age ≤ 12 years, OR 1.87 (1.23–2.85)
Jorgensen et al. (45)	Retrospective cohort	National registry/Denmark	4,400,000/7,017	Parity	↑ risk of RA with ↓ age at birth of first child (*p < * 0.001) Women with > 1 child at ↓ risk of RA vs. women with one: 2 children, RR 0.84 (0.78–0.90) 3 children, RR 0.83 (0.77–0.91)
Orellana et al. (46)	Case-control	Women aged 18–70 years, Sweden	4,946/2,035	Parity	Parity ↑ risk of ACPA-negative RA in ages 18–44, OR 2.1 (1.4–3.2) but not in ages 45−70 years ↑ risk of ACPA-negative RA with young age (<23 years) at first birth, OR 2.5 (1.5–4.1)
Ren et al. (47)	Meta-analysis	Women aged 15–84 years	2,497,580/11,521	Parity	Borderline ↓ risk of RA in parity vs. nulliparity, RR 0.90 (0.79–1.02)
Pikwer et al. (48)	Case-control	Women aged 44–74 years, Sweden	680/136	BF	Longer duration of BF ↓ risk of RA, OR 0.46 (0.24–0.91) if ≥13 months
Berglin et al. (49)	Case-control	Women aged 20–68 years, Sweden	350/70	BF	BF ↑ risk of RA, OR 4.8 (1.43–15.8)
Adab et al. (50)	Cohort	Women ≥50 years, China	7,349/669	BF	↓ risk of RA in BF, lower risk with increasing duration, OR 0.54 (0.29–1.01) if ≥ 36 months
Chen et al. (51)	Meta-analysis	Women aged 16–79 years	143,670/1,672	BF	↓ risk of RA in BF, OR 0.68 (0.49–0.92), dose response effect
Beydoun et al. (52)	Cohort	Women >60 years, USA	1,892/182	Menopause	↑ risk of RA with menopause <40 years vs. ≥50 years, OR 2.53 (1.41–4.53)
Bengtsson et al. (53)	Prospective cohort	Nurses Health Study, USA	237,130/1,096	Menopause	Early menopause (<44 years) ↑ risk of seronegative RA, HR 2.4 (1.5–4.0)
Merlino et al. (54)	Prospective cohort	Women aged 55–69 years	31,336/158	Menopause/ pregnancy/ misc	Age at menopause & age at last pregnancy ↓ risk of RA History of PCOS ↑ risk of RA
Salliot et al. (55)	Prospective cohort	Women aged 40–65 (at baseline), France	78,452/698	Multiple endogenous hormonal factors	Borderline ↑ risk of RA in early menarche (<13 years), HR 1.20 (0.9–1.5) Early age at 1st pregnancy (<22 years) ↑ risk of RA, HR 1.34 (1.0–1.7) Nulliparity ↓ risk of RA in non-smokers, HR 0.44 (0.2–0.8) ↑ risk of RA in early menopause (<45 years) in smokers, HR 1.54 (1.0–2.3)

RA, rheumatoid arthritis; RR, relative risk; BF, breastfeeding; ACPA, anti-citrullinated protein antibody; OR, odds ratio; HR, hazard ratio; PCOS, polycystic ovarian syndrome. ↑, increased; ↓, decreased.

**Table 2:** Epidemiological studies showing no association between endogenous sex hormones and RA pathogenesis.

**Reference**	**Design**	**Population/country**	**N (Total/RA)**	**Hormonal factor**	**Summary of findings**
Karlson et al. (43)	Prospective cohort	Nurses aged 30–55 (at baseline), USA	121,700/674	Parity	No effect of parity/age of first birth and risk of RA
Pikwer et al. (48)	Case-control	Women aged 44–74 years, Sweden	680/136	Parity	No effect of parity on risk of RA
Orellana et al. (46)	Case-control	Women aged 18–70 years, Sweden	4,946/2,035	Parity	No effect of parity/post-partum on risk of ACPA-positive RA
Chen et al. (56)	Meta-analysis	Women aged 15–79 years	2,385,179/13,374	Parity/pregnancy	No effect of parity, gravidity, pregnancy or post-partum on risk of RA
Orellana et al. (57)	Case-control	Women aged ≥18 years, Sweden	6,892/2,641	BF	No effect of BF on risk of RA
Salliot et al. (55)	Prospective cohort	Women aged 40–65 (at baseline), France	78,452/698	BF	No effect of BF on risk of RA
Beydoun et al. (52)	Cohort	Women >60 years, USA	1,892/182	Menopause	No effect of age at menarche and pregnancy history on post-menopausal RA

*ACPA, anti-citrullinated protein antibody; BF, breastfeeding*.

**Table 3:** Epidemiological studies of exogenous sex hormones and RA pathogenesis.

**Reference**	**Design**	**Population/country**	**N (Total/RA)**	**Hormonal factor**	**Summary of findings (95 % CI)**
Berglin et al. (49)	Case-control	Women aged 20–68 years, Sweden	350/70	OC	Use of OC ≥ 7 years ↓ risk of RA
Orellana et al. (57)	Case-control	Women aged ≥18 years, Sweden	6,892/2,641	OC	Ever use of OC ↓ risk of ACPA-positive RA, OR 0.84 (0.74–0.96) OC use > 7 years ↓ risk of ACPA-positive and ACPA-negative RA
Pedersen et al. (44)	Case-control	Women aged 18–65, Denmark	1,284/515	OC	OC ↑ risk of ACPA-positive RA, OR 1.65 (1.06–2.57)
Karlson et al. (43)	Prospective cohort	Nurses aged 30–55 (at baseline), USA	121,700/674	OC	No effect of OC use and risk of RA
Pikwer et al. (48)	Case-control	Women aged 44–74 years, Sweden	680/136	OC	No effect of OC on risk of RA
Adab et al. (50)	Cohort	Women ≥50 years, China	7,349/669	OC	No effect of OC on risk of RA
Chen et al.(58)	Meta-analysis	Women aged 16–84 years	221,022/4,209	OC	No effect on risk of RA but prevents progression to severe disease
Doran et al. (59)	Case-control	Women aged ≥18 years, USA	890/445	OC and HRT	↓ risk of RA with OC use, OR 0.56 (0.34–0.92), lower with first exposure in earlier years No association of HRT with risk of RA
Salliot et al. (55)	Prospective cohort	Women aged 40–65 (at baseline), France	78,452/698	OC and HRT	Nil effect of OC on risk of RA Nil effect of HRT on risk of RA in menopause ↓ risk of RA with oral progesterone use >24 months before menopause, HR 0.77 (0.6–0.9)
Salliot et al. (60)	Cohort	Early arthritis cohort, France	568	HRT	↓ risk of RA in women carrying HLA-DRB1 *01 and/or *04 alleles Protective effect of HRT on development of ACPA, OR 0.43 (0.24–0.77)
Orellana et al. (61)	Case-control	Women aged 18–75, Sweden	1,580/523	HRT	↓ risk of ACPA-positive RA in current users of HRT aged 50–59, OR 0.3 (0.1–0.8) ↓ risk of ACPA-positive RA in current users of combined HRT, OR 0.3 (0.1–0.7) but not estrogen-only HRT No association between HRT and ACPA-negative RA
Merlino et al. (54)	Prospective cohort	Women aged 55–69 years	31,336/158	HRT	↑ risk of RA with HRT, RR 1.47 (1.04–2.06)
Bengtsson et al. (53)	Prospective cohort	Nurses Health Study, USA	237,130/1,096	HRT	HRT use > 8 years ↑ risk of seropositive RA
Chen et al. (62)	Prospective cohort	Women aged ≥18 years with breast cancer, USA	190,620/4,460	Anti-oestrogens	↑ risk of RA with SERMs, OR 2.4 (1.9–3.0) ↑ risk of RA with AI, OR 1.9 (1.6–2.1)
Caprioli et al. (63)	Cohort	Women aged 57–74 with breast cancer, Italy	7,533/113	Anti-oestrogens	↑ risk of RA with AIs, HR 1.62 (1.03–2.56)
Wadstrom et al. (64)	Cohort/case-control	National registry, Sweden	95,362/15,356	Anti-oestrogens	No association between tamoxifen or AI and risk of RA

OC, oral contraceptive; HRT, hormone replacement therapy; ACPA, anti-citrullinated protein antibody; SERM, selective estrogen receptor modulator; AI, aromatase inhibitor; OR, odds ratio. ↑, increased; ↓, decreased.

### Endogenous sex hormones

#### Menarche

An abnormally early menarche was found to be associated with an increased risk of seropositive RA in a large prospective cohort study [relative risk (RR) 1.6 [95% confidence interval (CI) 1.1–2.4] for menarche <10 years] (43). This finding was supported by a more recent cohort study, although the association was weaker (HR) 1.20 for menarche <13 years (95% CI 0.9 - 1.5) (55). The data on menarche is conflicting, however, as a case-control study found an increased risk of anti-citrullinated protein antibody (ACPA)-positive RA with menarche at ≥15 vs. ≤ 12 years (44).

#### Pregnancy and post-partum

##### Hormonal changes in pregnancy

Pregnancy is associated with considerable increases in both estrogen (around four to six fold) and progesterone (around three to eight fold) (65). These hormones rise progressively through pregnancy, peaking at the third trimester and falling within weeks post-partum.

##### Pregnancy, parity and risk of RA

In an initial small case-control study, pregnancy was associated with a reduced risk of RA onset which did not reach statistical significance [odds ratio (OR) 0.3, 95% CI 0.04–2.6], while there was an increased incidence of RA during the first 3 months post-partum (OR 5.6, 95% CI 1.8–17.6) (66). Similar results were found in another early case-control study (67). The risk of RA onset has been shown to persist for the first year (OR 3.8, 95% CI 1.45–9.93) (68) and up to 24 months post-delivery (incidence rate ratio 1.7, 95% CI 1.11–2.70) (69).

In early studies, nulliparity was associated with a roughly two-fold risk of RA onset (70), but modern studies have shown conflicting findings regarding parity and risk of RA. Some studies have found that having more than one pregnancy increased the risk of RA (46), particularly with young age at first pregnancy (45, 46), while others have found a protective effect of parity (45, 47, 71). One retrospective cohort study found a dose-response relationship between the protective effect of parity and risk of RA (45), but this was not replicated in a later meta-analysis (47). The most recent systematic review found no association between gravidity and parity and the risk of developing RA (56).

##### Pregnancy and RA disease activity

RA was first observed to spontaneously remit in pregnancy in the 1930s (72). Historical studies reported that up to 90% of patients improved during pregnancy, but this effect is less apparent with modern treatment regimens (3, 73). As safety data regarding the use of traditional and biologic disease modifying drugs in pregnancy accumulates (74, 75), increasing numbers of patients maintain low disease activity or remission through pregnancy with a treat-to-target strategy (76).

Recent systematic reviews of the potential mechanisms of RA disease improvement in pregnancy have not identified any human studies that have specifically investigated the impact of rises in estrogen or progesterone on alterations of RA disease activity in pregnancy (77, 78).

#### Breastfeeding

In an early study, breastfeeding was associated with post-partum flare in inflammatory polyarthritis; this finding led the authors to suggest that prolactin may be responsible for this phenomenon (79). Since then, duration of breastfeeding has been most consistently associated with a decreased risk of RA (48, 50, 51). Other case-control (57) and large prospective cohort (43, 55) studies have found no significant association between breastfeeding and risk of RA, while a single study identified an increased risk (49). The data regarding the role of prolactin in human RA is limited and conflicting (80), but in a murine model of collagen-induced arthritis, treatment with bromocriptine, an inhibitor of prolactin secretion, suppressed post-partum flare (81).

#### Menopause

A consistent finding is the increased risk of RA in early menopause (52, 82, 83). In the large Nurses Health Study cohort, menopause at <44 years increased the risk of seronegative RA [hazard ratio (HR) 2.4, 95% CI 1.5–4.0] (53). The menopause has also been associated with the development of ACPA in first degree relatives of patients with RA (84).

#### Androgens

In men with RA, low serum levels of testosterone were found to be strongly predictive of seronegative disease (OR 0.31, 95% CI 0.12–0.85) but not significantly predictive of seropositive disease (85). Men with untreated hypogonadism have been found to be at increased risk of a range of autoimmune diseases, including RA (HR 1.31, 95% CI 1.22–1.44) (86), as are men with Klinefelter syndrome (RR 3.3, 95% CI 2.0–5.2)(87).

### Exogenous sex hormones

#### Oral contraceptives

There are conflicting reports on the effect of oral contraceptives (OC) on the risk of RA.

Early reports suggested a beneficial effect, with a case-control study (*n* = 115) showing lower current use of OC in new cases of inflammatory polyarthritis (OR 0.22, 95% CI 0.06–0.85) (88). A further case control study found that OC use roughly halved the risk of RA, with stronger protection from earlier OC preparations (59). A matched case-control study found, conversely, an increased risk of ACPA-positive RA with OC use (44). Most recent reports, however, show no such effect (43, 50, 58, 89), although a meta-analysis has suggested that OC may reduce the risk of progression to severe disease in established RA (58). Two studies have suggested that extended OC use (>7 years) may protect against RA (49, 57). The differing findings regarding OC use and risk of RA have been suggested to be due to a lowering of their estrogen content over time (59, 90).

#### Hormone replacement therapy

Use of hormone replacement therapy (HRT) was associated with a significantly elevated risk of RA in a large prospective study of postmenopausal women (RR 1.47, 95% CI 1.04–2.06) (54). Conversely, a case-control study found a reduced risk of ACPA-positive RA in women aged 50–70 years who were current users of combined (estrogen plus progestogen) HRT (OR 0.3, 95% CI 0.1–0.7) (61). This finding supported that of an earlier cohort study which found a protective effect of HRT on the development of ACPA in early arthritis (60). Other studies have found no association between HRT use and risk of RA (59).

#### Anti-estrogen agents

Selective estrogen receptor modulators (SERMs) and aromatase inhibitors (AI) are used for the treatment of breast cancer. SERMs competitively inhibit estrogen binding to its receptor and may have agonistic or antagonistic properties in different target tissues, while AI reduce endogenous production of estrogen. A large national database study found an increased incidence of RA with use of either of these agents (OR 2.4 for SERMs (95% CI 1.9–3.0) and 1.9 (95% CI 1.6–2.1) for AI, each with >12 months treatment) (62). In another large population-based study, the use of aromatase inhibitors to treat breast cancer was associated with an increased risk of RA (adjusted HR 1.62, 95% CI 1.03–2.56) (63). In contrast, a recent large national registry study found no increased risk of RA with either tamoxifen (a SERM) or AI (64).

## Clinical trials of sex hormones in RA

### Estrogen and progesterone

Investigations into the therapeutic potential of sex hormones in RA have been disappointing. A randomized placebo-controlled trial of adjuvant estrogen therapy at physiological doses compatible with pregnancy in postmenopausal female RA patients was negative (91). Randomized controlled trials of ERα (92) and ERβ (93) agonists in RA were both negative. Large randomized control trials of HRT (either estrogen or estrogen and progesterone combined) in postmenopausal women with RA failed to demonstrate any benefit on symptom severity over placebo (94).

Interestingly, however, the data emerging from the field of multiple sclerosis, another disease that improves in pregnancy, is more encouraging regarding the potential therapeutic benefit of estrogen: a placebo-controlled phase 2 trial of estriol met its endpoint of a significant reduction in relapse rate (95). Progesterone or its synthetic derivatives have not been studied in clinical trials as a treatment option in RA.

### Androgens

Well-designed trials of supplemental androgen therapy in patients with RA are lacking. Two preliminary studies of treatment with testosterone in the 1990s suggested positive results in male (*n* = 7) (96) and postmenopausal female (97) patients (*n* = 107). Meanwhile, two trials of dehydroepiandrosterone (DHEA) have been published. A small open-label trial of DHEA treatment in elderly RA patients (six post-menopausal female and five male) found no benefit on disease activity (98), while a randomized placebo-controlled trial of DHEA in pre-menopausal RA patients (*n* = 46) found improvements in quality of life but not disease activity scores (99).

## Discussion

Despite extensive work in both basic laboratory and clinical studies, the exact impact of sex hormones on RA pathogenesis remains controversial. The epidemiological data described above is conflicting, with both high and low estrogen/progesterone states being found to be protective or risk factors, or to have no effect on development or progression of RA, in different studies. Namely, age of menarche, age of menopause, parity status, breastfeeding, use of the oral contraceptive, and HRT have all been found by different studies to have opposing effects on the risk of RA. Meta-analyses pertaining to parity and risk of RA found only a borderline, or zero, association (47, 56), as did that regarding the oral contraceptive (58). In contrast, the studies relating to breastfeeding have been more consistent, in that only one case-control study found an increased risk of RA with breastfeeding (49), while several others have found a protective effect, including four reporting a dose-response relationship (43, 48, 50, 51). These discrepancies are likely explained to an extent by heterogeneity in study design, variations in study populations and definitions of reproductive variables (e.g., early menarche), and inherent limitations in case-control studies such as recall bias and lack of consideration of confounding variables. On a biological level, many environmental and genetic factors may influence sex steroid signaling *via* their intracellular receptors, which may be another reason for the conflicting data from epidemiological studies.

Despite discrepancies in the published literature, several patterns do emerge. The most consistent findings are the increased risk of RA at early menopause and post-partum, and decreased disease activity in pregnancy. Therefore, declining estrogen and/or progesterone levels (in post-partum and menopause) are consistently linked to the onset of RA, while high levels of these hormones are protective during pregnancy (although many other factors may be relevant to reduced disease activity in pregnancy, as we and others have previously noted).

While there is an abundance of epidemiological data regarding reproductive factors and risk of RA, one possible avenue of research which, to our knowledge, has not been explored is the study of individuals with gender dysphoria [except a single case report (100)]. For instance, it would be intriguing to study whether the risk of RA in transgender individuals is modified by gender affirming therapy, or whether disease activity of pre-existing RA is altered by such treatments. This topic is discussed in detail elsewhere in this chapter.

There is a wealth of basic scientific data demonstrating the immunomodulatory actions of oestrogens, progestogens and androgens. The therapeutic potential of these hormones for treating RA suggested by results from animal models has to date not translated into successful clinical trials. In humans it is likely that there is a much more complex interaction between sex hormones and a multitude of genetic and environmental risk factors (e.g., smoking, obesity, alcohol consumption) for RA. Progestogens and androgens both exhibit more broadly anti-inflammatory actions than those of oestrogens and it is interesting to note the recent finding that perimenopausal oral progestogen use reduced the risk of RA (55) and that combined HRT, but not estrogen alone, strongly reduced the risk of ACPA-positive disease (61). However, neither of these hormones have been evaluated in large well-designed trials of RA, perhaps due to concern regarding the potential side effects of systemic administration.

It is important to point that there are other sex-related factors which have been proposed as being of potential importance in RA pathogenesis that we have not considered in the present review, including microchimerism (101), sex chromosomes (102) and sex differences in gut microbiota (103). However, none of these other factors have to date shown such direct links with RA disease onset and/or progression.

In terms of future study, the rapidly expanding field of high throughput multiomics technologies (e.g., genomics, transcriptomics, proteomics and metabolomics), particularly at the single cell level, is starting to dissect the pathobiological basis of clinical heterogeneity in human disease, including in RA (104). With both epidemiological and *in-vitro* studies proving ultimately insufficient to unravel the relationship between sex hormones and RA pathogenesis, the application of these novel techniques are a tantalizing proposition for investigators in this area.

## Conclusion

Sex hormones are immunomodulatory with pleiotropic effects on the immune system. There are conflicting reports regarding endogenous and exogenous sex hormones and RA pathogenesis, but declining estrogen levels in the menopause and post-partum are consistently associated with an increased risk and severity of RA. These findings, however, have not translated into improved therapies in RA, although progesterone and androgens warrant further evaluation as potential therapeutic agents in clinical trials.

## Author contributions

CR and IG conceived of the article. CR performed the literature search, reviewed the source papers, and drafted the manuscript. IG independently performed the search, contributed to inclusion/exclusion of source literature, and edited the final manuscript. All authors contributed to the article and approved the submitted version.

## Funding

CR received support from the British Society of Rheumatology (BSR CID-2146992), the Rosetrees Trust (M707), University College London, and an Investigator Initiated Studies grant from UCB Biopharma SRL (UCB: IIS-2019-134708). IG has received speaker's fees and travel fees from UCB. The funders were not involved in the study design, collection, analysis, interpretation of data, the writing of this article or the decision to submit it for publication.

## Conflict of interest

The authors declare that the research was conducted in the absence of any commercial or financial relationships that could be construed as a potential conflict of interest.

## Publisher's note

All claims expressed in this article are solely those of the authors and do not necessarily represent those of their affiliated organizations, or those of the publisher, the editors and the reviewers. Any product that may be evaluated in this article, or claim that may be made by its manufacturer, is not guaranteed or endorsed by the publisher.
